# Isolated Trigeminal Mononeuropathy: A Possible COVID-19 Sequelae

**DOI:** 10.7759/cureus.68522

**Published:** 2024-09-03

**Authors:** Hamza Jamil, Momin Noor, Jared Hollinger, Sydney Martin, Danial Bajwa, Syed Hashim Ali Inam

**Affiliations:** 1 Neurology, Army Medical College, Rawalpindi, PAK; 2 Neurology, Marshall University Joan C. Edwards School of Medicine, Huntington, USA; 3 Neurology, Combined Military Hospital, Rawalpindi, PAK

**Keywords:** acute pain, painful trigeminal neuropathy, post-covid sequelae, trigeminal mononeuropathy, trigeminal sensory loss

## Abstract

COVID-19 infections have been linked with multiple neurological manifestations. One of the infrequent complications of post-COVID-19 infection is trigeminal neuropathy. Despite its infrequency, few cases of trigeminal neuropathy following COVID-19 infection have been documented in the literature. However, there remains a paucity of evidence regarding isolated trigeminal neuropathy following COVID-19, particularly in cases devoid of pain but characterized by sensory deficits such as loss of sensation and paresthesia only. We describe a clinical case of trigeminal neuropathy that emerged after a COVID-19 infection at our institution. Our case report delves into the clinical presentation of such trigeminal neuropathy that would aid clinicians in including this pathology in their differential diagnosis.

## Introduction

The COVID-19 pandemic has not only presented an unprecedented global health crisis but has also unveiled a myriad of neurological manifestations, including various cranial neuropathies [1]. The literature documents the occurrence of unilateral and bilateral cranial neuropathies associated with COVID-19 infection, ranging from facial nerve palsy to optic neuritis [2]. Among these neurological complications, trigeminal neuropathies have been reported, albeit less frequently, compared to other cranial nerves [3]. There have been reports of COVID-19 infections leading to transverse myelitis, autoimmune encephalitis, Guillain-Barré syndrome (GBS), and chronic infectious demyelinating polyneuropathy [4]. There have been reports of overlap syndrome where COVID-19 infection led to an overlap of transverse myelitis and GBS [5-7].

In this paper, we present a unique case report of isolated trigeminal neuropathy (TNO) following COVID-19 infection, where the patient manifests no overt pain but presents with sensory disturbances, including loss of sensation and paresthesia. Our case contributes to the limited body of literature exploring the association between COVID-19 and trigeminal neuropathies, shedding light on an uncommon manifestation that warrants attention in the context of post-infectious neurological complications.

Through a detailed examination of the clinical presentation, diagnostic evaluation, and management strategies employed in our case, we aim to elucidate the clinical presentation of TNO following COVID-19 infection. Furthermore, we underscore the importance of heightened vigilance among clinicians to recognize and appropriately manage such atypical neurological sequelae in individuals recovering from COVID-19. By delineating the clinical course and outcomes of this rare presentation, we hope to enhance understanding, facilitate early detection, and optimize management strategies for isolated TNO following COVID-19 infection. This case report underscores the necessity for continued vigilance and research efforts to unravel the intricate interplay between COVID-19 and neurological complications, thereby improving patient care and outcomes in the post-pandemic era.

## Case presentation

A 64-year-old white male was referred from the ophthalmology clinic because he was found to have an absence of corneal reflex on examination. In the neurology clinic, he was found to have chronic numbness and tingling sensations on the left side of his face, left-sided facial numbness, and paresthesia that “waxes and wanes,” particularly involving the left perioral region, along with cloudy vision and persistent dysgeusia. The patient also reported hyposmia and hypogeusia. Of note, the patient reported having tested positive for the SARS-CoV-2 virus just prior to the onset of symptoms.

**Figure 1:**
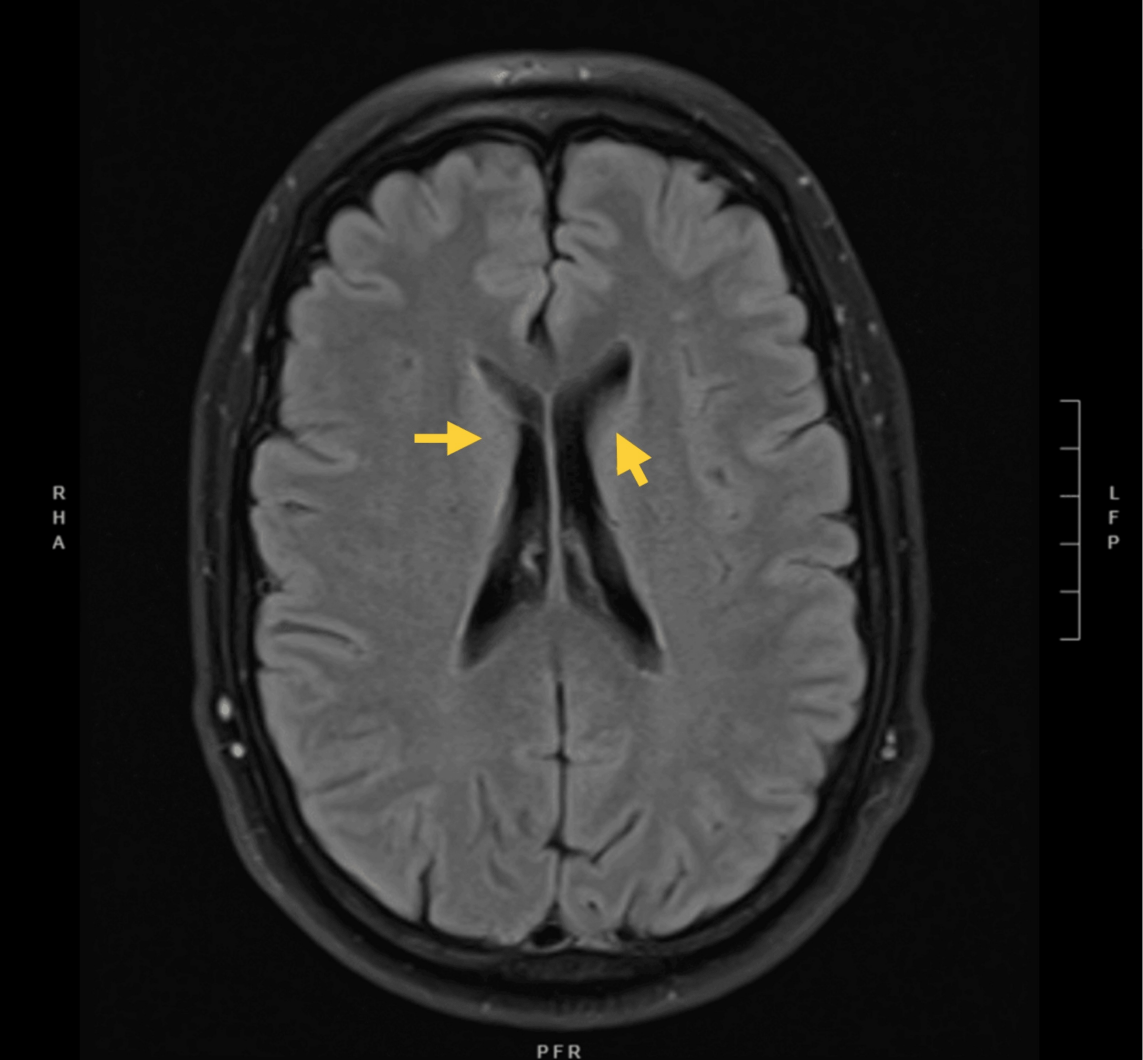
T2 fluid attenuated inversion recovery (FLAIR) MRI sequence showing several small white matter FLAIR hyperintensities around ventricles likely secondary to chronic small vessel ischemic disease (yellow arrows).

The initial evaluation included magnetic resonance imaging (MRI) without contrast, which revealed several small white matter flair hyperintensities, which were deemed age-appropriate, as shown in Figure 1.

Symptoms had developed gradually over a period of six months, prompting further investigation. Physical examination revealed left hemi-facial numbness with partial sensory loss of temperature and pinprick sensation in the distribution of the trigeminal nerve (cranial nerve V). Sensation seemed to be more diminished in V2 > V1 > V3 distribution of the trigeminal nerve. The patient denied the presence of pain and, as such, was not appreciated during the encounter. No obvious weakness of the muscles of mastication or facial droop was observed. Other mild abnormalities on his neurologic exam were chronic and secondary to lumbar spinal disease. The review of systems was otherwise negative. Diagnostic workup included Lyme serology, measurement of angiotensin-converting enzyme (ACE) levels, and evaluation of vitamin B6, folate, and vitamin B12 levels. Additionally, a thyroid panel, syphilis serology, C-reactive protein (CRP), erythrocyte sedimentation rate (ESR), a barium swallow study, and electromyography/nerve conduction studies (EMG/NCS) focusing on blink reflexes were conducted. The diagnostic workup was negative and unrevealing, except for an abnormality in the nerve conduction study.

**Figure 2:**
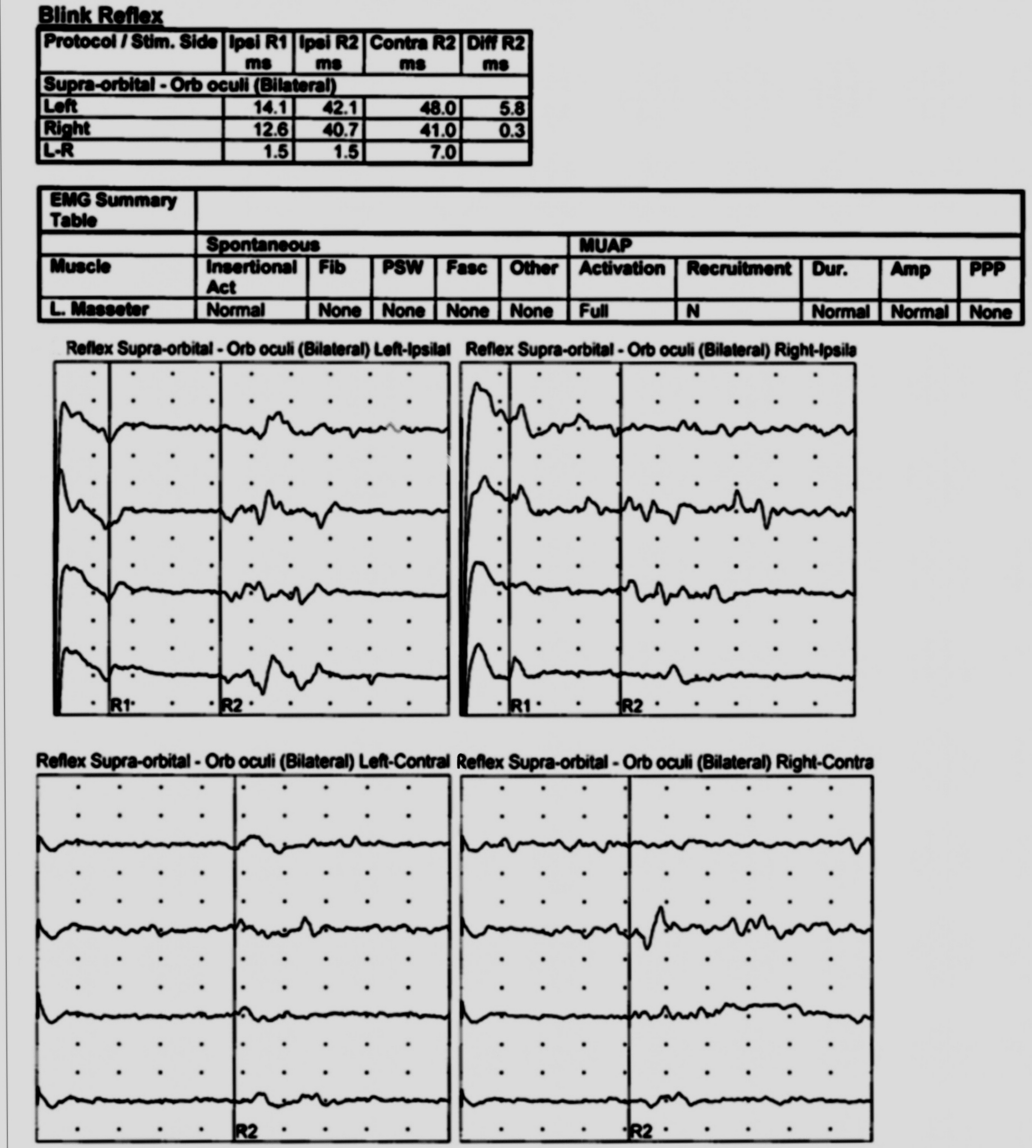
NCS showed blink reflexes performed bilaterally, demonstrating normal responses stimulating on the right with easily obtainable R1, ipsilateral, and contralateral R2 responses. Stimulating on the left, a clear R1 waveform was not readily obtainable. Ipsilateral and contralateral R2 reflexes were generally present but with slightly prolonged latency relative to the right. EMG of the left masseter was normal. Amp: amplitude, Contra: contralateral, Dur: duration, EMG: electromyography, Fib: fibrillation, Fasc: fasciculation, Ipsi: ipsilateral, L: left, N: normal, NCS: nerve conduction study, Orb: orbicularis, PPP: polyphasics, PSW: positive sharp wave, R: right, S: sharp, PSW: positive sharp wave

NCS findings were consistent with an incomplete left trigeminal mononeuropathy. Ipsilateral and contralateral R2 responses (when stimulating on the left) were delayed relative to the right, which indicated a demyelinating component, and though there was no clearly obtainable R1 on the left, afferent pathways were at least partially intact, allowing for bilateral R2 responses. EMG of the masseter muscle did not reveal any notable abnormalities. NCS of supraorbital muscles with respect to blink reflex showed a decrease in velocities on the left side compared to the right. This is seen in Figure 2 as follows.

On his follow-up visit to the clinic three months after his first visit and one year after the development of symptoms, the patient reported persistent left facial numbness, dysgeusia, hypogeusia, and hyposmia. Additionally, the patient described a “gritty” feeling in his left eye, possibly due to decreased lacrimation. This could be explained by the dysfunction of the lacrimal glands that are innervated by the lacrimal nerve, the smallest branch of the ophthalmic nerve, which itself is a branch of the trigeminal nerve. Additionally, decreased corneal sensation with TNO could result in a reduced sensation for foreign bodies in the eye. Intriguingly, the patient now did report occasional and intermittent burning neuropathic pain.

Suspected TNO secondary to SARS-CoV-2 infection was considered. Definitive diagnosis could only be made on autopsy and inspection of the trigeminal ganglion, but diagnosis in this case was made on electro-diagnostic testing.

We suspected the symptoms would remain stable or improve over time as the nerve heals. The patient declined symptomatic treatment as of now and wanted to monitor his symptoms and follow up with neurology.

## Discussion

TNO is a dysfunction of the motor and sensory components of the trigeminal nerve [8,9]. The differential diagnoses include trigeminal neuralgia (TN), in which pain is paroxysmal, abrupt in onset and termination, and without motor weakness [10,11].

The other differentials include trigeminal autonomic cephalalgias (TACs) such as SUNCT (short‐lasting unilateral neuralgiform headache attacks with conjunctival injection and tearing) and SUNA (short‐lasting unilateral neuralgiform headache attacks with cranial autonomic symptoms) whose main characteristics are being accompanied by autonomic symptoms (tearing/redness of the eyes; stuffy/runny nose; eyelid swelling on the same side as the headache; sweating of head and neck area; redness of head and neck area; ear fullness and discomfort; droopy eyelid or pinpoint pupil), and our patient had none of these [12,13].

The pathophysiology of TNO due to COVID-19 is unclear as of now, but there are hypotheses of direct invasion via binding to receptors on ACEs, which are present in trigeminal nerves. Some researchers say it is indirect invasion by cytokine release, but more case reports and extensive research are needed to understand the pathophysiology of COVID-19 causing TNO [14,15]. There are gaps in understanding of the neurotropic nature of the COVID-19 virus, so further research and cases are needed to dive into the pathophysiology, diagnostics, and possibly the treatment/management of TNO [12-15]. The coronavirus disease outbreak caused by SARS-CoV-2 has become a global health problem. COVID-19 is a highly infectious and multifaceted disease, and already millions of people have been infected worldwide. According to the literature, the COVID-19 pandemic caused a major reduction in emergency surgical operations as well as overall admissions to emergency departments because of the widespread hospital fear and anxiety experienced by most patients during the peak of this outbreak [16]. Per recent studies, butyrylcholinesterase (BChE), a non-specific cholinesterase enzyme, has been correlated with the risk of hepatic dysfunction progression and, more recently, infectious diseases and septic shock [17]. This could hint toward a possible new biomarker behind the COVID-19-related pathology [17].

Our study highlights only one case, but it can pave the way for further research on the topic so clinicians can manage TN in the context of COVID-19. The limitations of the study are that it was a clinical diagnosis with no clear understanding of the pathophysiology of how COVID-19 can cause TNO.

## Conclusions

There is a need to better understand the neurotropic nature of the COVID-19 virus as it is involved in multiple neurological manifestations. The true mechanism behind the pathology of COVID-19 leading to neurological disease still remains unknown. Considering the prevalence of COVID-19 infection, it is necessary to emphasize and discuss the variable presentation of COVID-19 infections and their aftereffects. We discussed a presentation of post-COVID-19 TNO who presented to our institution. It is necessary to inquire about recent COVID-19 infection in such presentations to avoid misdiagnosis. Further research and cases are needed to dive into the pathophysiology, diagnostics, and possibly the treatment/management of the TNO. This would help clinicians add the diagnosis of TNO to their differentials. Our study highlights only one case, but it can pave the way for further research on the topic so that clinicians can manage trigeminal nerve-related pathologies in the context of COVID-19.
